# Outcomes and experiences of music workshops for adolescents with depression and anxiety: An exploratory noncontrolled trial in Bogotá

**DOI:** 10.1186/s13104-024-07007-z

**Published:** 2024-12-02

**Authors:** Carlos Gómez-Restrepo, María Camila Roldan, Karen Ariza-Salazar, Natalia Godoy-Casasbuenas, Catherine Surace Arenas, Paul Heritage, José Miguel Uribe-Restrepo, Catherine Fung, Stefan Priebe

**Affiliations:** 1 Department of Clinical Epidemiology and Biostatistics, Pontificia Universidad Javeriana, Bogotá, Colombia; 2 Department of Psychiatry and Mental Health, Pontificia Universidad Javeriana, Bogotá, Colombia; 3 Hospital Universitario San Ignacio, Bogotá, Colombia; 4 Fundación Nacional Batuta, Bogotá, Colombia; 5 School of English and Drama, Queen Mary University of London, London, UK; 6 Unit for Social and Community Psychiatry (WHO Collaborating Centre for Mental Health Service Development), Queen Mary University of London, London, UK; 7 East London NHS Foundation Trust, London, UK

**Keywords:** Mental health, Adolescents, Depression, Anxiety, Musical learning

## Abstract

**Objective:**

Adolescents frequently experience mental distress. However, there is little research on community activities that help adolescents recover from depression and anxiety. This study investigated the outcomes and experiences of music workshops for helping adolescents overcome anxiety and/or depression.

**Results:**

Thirty-four participants aged 15 and 16 years were recruited from a cohort study of adolescents with symptoms of anxiety and/or depression and invited to participate in a musical education and practice workshop consisting of five weekly sessions. At the beginning and end of the workshops, symptoms of anxiety and depression were self-rated on the GAD-7 and PHQ-8. Experiences were assessed on a brief questionnaire with open-ended questions. The average attendance of groups was 86%, but only 56% of participants attended all five sessions of the workshops. The symptom levels did not significantly change during the intervention. Experiences were largely positive. Participants appreciated the interactions in the group and the learning of new skills. Some criticized the workshops as too short and found the logistics of attending difficult.

**Trial registration:**

Current Controlled Trials ISRCTN50583823. Date Applied 18/03/2022.

**Supplementary Information:**

The online version contains supplementary material available at 10.1186/s13104-024-07007-z.

## Introduction

According to the WHO, depression and anxiety are among the leading causes of illness and disability among adolescents [1, 2]. Adolescence is characterized by multiple physical changes and social difficulties and can be associated with increased vulnerability to mental health problems [3, 4], as recently demonstrated during the COVID-19 pandemic [5–8].

In Colombia, the National Mental Health Survey of 2015 showed that approximately 7.2% of adolescents have symptoms of anxiety or depression [9]. Studies conducted during the COVID-19 pandemic in Colombia suggest an even greater prevalence of such symptoms [10–14]. Consequently, adolescent mental health is a priority for the national health agenda [3, 15], and there is a need to identify interventions that help adolescents overcome mental distress in the form of depression and anxiety.

Several studies suggest that arts-based programs can have a positive impact on young people’s health, psychological well-being, and resilience. Arts can be seen as a resource for dealing with challenging situations and as a tool for developing personal strengths and skills [16–18]. Hence, participation in artistic activities might help adolescents who struggle with mental health problems. A meta-analysis revealed that adolescents who received music-based therapies experienced significant reductions in symptoms of depression and anxiety [19].

Given the importance of improving the mental health of adolescents, interventions that are available in the community and effective at reducing mental distress need to be developed and tested. This is particularly important in low- and middle-income countries such as Colombia, where there are fewer resources for conventional clinical services than in many high-income countries.

Therefore, the objective of this study was to evaluate the outcomes and experiences of musical workshops in an urban population of high school adolescents with symptoms of depression or anxiety.

## Methods

### Study design

We carried out a noncontrolled trial (with mixed methods design) in Bogotá, Colombia, within the OLA Research Program (Building resilience and resources to reduce depression and anxiety in young people from urban neighbourhoods in Latin America) [20]; The OLA Research Program is a multicentre research program focused on exploring the resources, activities and attitudes that help young people prevent and recover from mental distress in three large Latin American cities [20]; This objective is expected to be achieved through the development of different activities (Phases) including the formation of a cohort of young people with symptoms of emotional distress who will be followed up for a period of two years.

#### Study setting

The workshops were provided in two public schools in the city of Bogotá: INEM Santiago Perez and IED Atanasio Girardot. Those schools collaborated with Javeriana University in the OLA Research Program and allowed us to use the school facilities for this study, regardless of whether the adolescents in this study belonged to those schools or not.

The workshops were not part of the usual school day, they were provided as extra-curricular activities for a small group of participants of the OLA Cohort who came from different schools in Bogota (Colombia).

#### Participants

Two music teachers and employees of Fundación Nacional Batuta designed and developed the program of the music workshop [21]. 

The inclusion criteria for the participating adolescents were as follows: (a) 15–16 years old when they were recruited to the OLA cohort; (b) symptoms of anxiety and/or depression at the beginning of the OLA Program and at 6 months of follow-up (the threshold was defined as a score of 10 or more on the Patient Health Questionnaire-8 (PHQ-8) [22] and/or the Generalized Anxiety Disorder Assessment Questionnaire-7 (GAD-7) [23]; (c) ability to give assent; and (d) informed consent from the parent/guardian.

The exclusion criteria were (a) already regularly participating in a music band/orchestra or having advanced musical abilities (based on an initial assessment) or (b) no interest in participating in any artistic activity (as seen in the OLA baseline assessment and checked by phone).

#### Recruitment

The research team identified 177 potential participants among those adolescents in the Colombia OLA Research Program cohort who participated in the 6 months follow-up or were close to participating (because their contact window was close to opening). Of these, 48 adolescents were not screened because some of them were participants without symptoms at OLA´s 6MFU, had pending 6-month follow-up, or were unable to contact.

Two steps in the initial contact with the remaining 129 potential participants were made via telephone: First, participants were contacted to assess other exclusion criteria (such as advanced music skills or lack of interest in the arts). Subsequently, as soon as the logistical details of the study were defined (dates and times, locations), the 81 eligible participants were contacted to assess their interest in participating in a music workshop and availability.

**Fig. 1:**
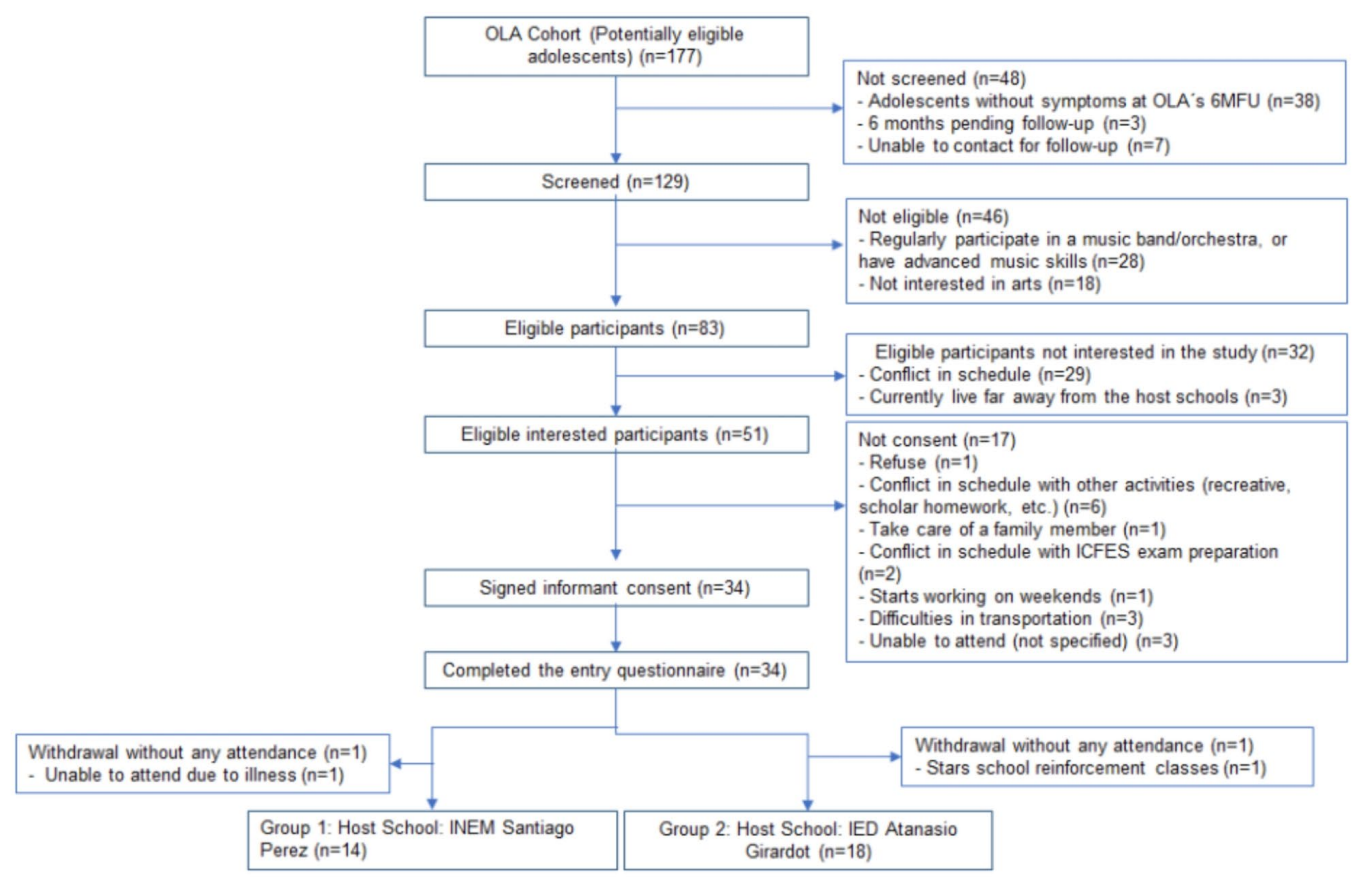
Recruitment process for the study

After the initial contact, 51 eligible participants interested in participating in the study were identified with whom the informed consent/assent process was conducted. The informed consent/assent process was conducted remotely by telephone with the adolescents and their parents or legal guardians, who sent a photo or scan of the signed document electronically. Participants were allocated to one of two musical workshops, depending on how close they lived to one of the two schools. Figure 1. Describes the recruitment process of the study.

#### Music workshops

During May and June 2022, two music workshops were carried out face-to-face at the same time, with a total duration of approximately 10 h, distributed in five weekly sessions per workshop. Each session took place first in one group/school and then in the other group/school. (the workshop leaders travelled between the two schools each session, for a period of 30 min)

The participants received initial group training in singing and playing the xylophone and other percussion instruments called “plates” (that were provided by the study) that allow the discovery of the singing voice, rhythm, listening and creative and musical capacity. In addition, in one session, they participated in the collective construction of new verses from an original song of Fundación Batuta and performed a brief closing show demonstrating the learned skills during the last session, open to some family and friends.

The design of the workshop was made in accordance with Batuta´s intervention model which focuses on: (a) work from musical practice through learning and playing musical instruments and singing, (b) privilege collective musical practice, (c) promote a state of here and now, where concentration and commitment are exercised and (d) promote decision-making situations with scenarios of consensus and dissent.

#### Data collection

Basic sociodemographic information (age, gender, locality of residence, school in which participants currently studied) was collected through the OLA cohort study log and confirmed through telephone contact with each participant.

Symptoms of depression and/or anxiety for this study were self-rated on the PHQ-8 and GAD-7 at two-time points: before the first workshop session (entry questionnaire) and immediately after the last session or within a week of the last session (exit questionnaire), respectively. These questionnaires were completed virtually via Research Electronic Data Capture (REDCap) with online contact with a researcher or in person through a paper questionnaire, with subsequent data entry to the REDCap platform.

Logistical information about the development of each workshop session (attendance and duration) was collected through a brief paper form, which was completed and uploaded to REDCap by a member of the research team.

Participants’ experiences of the music workshops were collected in the participants’ exit questionnaire with three open-ended questions regarding the aspects they liked most and least about the music workshop, as well as potential benefits of the workshop perceived by the participants.

### Data analysis

Differences in symptom variables between the entry and exit scales were calculated using the R programming language [24] and the RStudio interface [25] through a t-test with two tails, and uneven variance was applied, with a p value set at 5%. Of the original 30 participants who completed the exit questionnaire, we excluded five who scored 9 or less on both scales at the entry questionnaire since they did not present with symptoms of depression or anxiety for this study. We then conducted a sensitivity analysis including these five participants.

Qualitative data obtained in the exit questionnaire were analysed using content analysis [26]. Two independent members of the research team familiarized themselves with the transcribed responses and separately developed an open or inductive coding, aimed at defining the representative codes for the content related to the adolescents’ experiences of the workshop, Subsequently, these codes were discussed, and patterns were compared and identified (including the number of respondents having mentioned each code) by the research group that allowed the interpretation of the meanings. Discrepancies were discussed with the research team until consensus was reached.

## Results

A total of 34 adolescents who met the inclusion criteria were invited to participate, signed the informed consent form, and were enrolled in the study (Fig. 1).

We had two withdrawals of participants without any attendance (because of personal or health reasons). Two further participants did not complete the exit questionnaire because they only attended one session (one because the participant had competing commitments and one because he had no interest in continuing). Therefore, the final sample of this study included 30 participants, 25 of whom had symptoms above the threshold at the beginning of the workshops.

A total of 32 adolescents (23 female, 9 male) were allocated to the two musical workshop groups, 14 (44%) in INEM Santiago Perez and 18 (56%) in IED Atanasio Girardot. Overall, 86% of the participants (an average of 27.6 participants out of 32 total participants) attended each session of the music workshops and 56% of the adolescents (18 participants) attended all of the scheduled workshop sessions (five sessions). (see attendance tables in supplementary material)

There was no significant change in symptoms of depression or anxiety during the intervention (Table 1). Additionally, the sensitivity analysis we carried out did not substantially alter the findings of the main analysis (Table 2).

**Table 1:** Symptoms before and after workshops

	*n*	Before (mean)	SD	After (mean)	SD	t	*p*
*GAD-7*	*25*	*13.08*	*4.49*	*12.24*	*4.23*	*1.18*	*0.25*
*PHQ-8*	*25*	*13.36*	*4.48*	*13.20*	*4.63*	*0.16*	*0.88*

**Table 2:** Symptoms before and after workshops of all participants including those with symptoms below the threshold at the beginning of the workshops

	*n*	Before (mean)	SD	After (mean)	SD	t	*p*
*GAD-7*	*30*	*12.03*	*4.77*	*11.73*	*4.35*	*0.44*	*0.66*
*PHQ-8*	*30*	*12.23*	*4.88*	*12.23*	*4.88*	*-0.51*	*0.61*

What the participants reported having liked the most about the workshops was ‘interacting with others’ (referred by 20 participants), which included codes as ‘meeting new people’ and ‘socializing with others’. Thirteen participants reported that they ‘enjoyed’ the workshop with codes related with ‘Enjoyment of musical activities’ and ‘Enjoyment of the workshop space’. Eleven participants stated other aspects related with ‘Wellbeing’, with codes such as ‘rupture with daily routine’, ‘getting out of the comfort zone’, ‘having a safe space/wellness environment’, ‘being relaxed’, ‘being concentrated’ and ‘gaining in self-confidence/self-security’.

*“I loved that I was able to meet and socialize with people my age*,* I truly needed it*,* for me it was a relief from everyday life*,* I would have liked more sessions. Additionally*,* the musical part was calming in part… I truly had a great time socializing.” (Female*,* 16 years old)*.

They also appreciated the positive attitudes of the teachers:

*“We could express ourselves freely without fear of being judged*,* and the teachers gave us a lot of peace” (female*,* 15 years old).*

Adolescents disliked the short duration of the workshop programme and of each workshop session (13 participants). Eight participants disliked that they could ‘access only a few instruments’, had ‘difficulties in the learning process’ and found some activities ‘too long or repetitive’.

*“The short duration and that I would have liked it to be for a longer period*,* to have spaces to be able to socialize with my colleagues.” (Female*,* 15 years old)*.

The perceived benefits focused on positive impacts on their well-being (20 participants) and the learning of musical skills (14 participants). The effects on well-being included ‘Free the mind/distraction’, ‘rupture with the daily routine’, ‘loss of fear/insecurity’, ‘less anxiety or more relaxation’, and ‘control of repressed feelings’. Participants also reported having perceived the workshop as a ‘well-being space’, meaning that they felt good during the activities and perceived that their mood was improved or that depressive symptoms were reduced.

“*Perhaps my depression lowered a little*,* having talked and met new personalities helped me a lot with feeling so bad*,* plus I did not have to think so much because I was not* alone” *(Female. 16 years old)*.

## Discussion

We tested a relatively novel intervention in the form of brief music practice workshops. Workshop adherence was reasonable, especially considering that many young people discontinue verbal psychotherapy early, even before they have attended four sessions [27]. Moreover, data were collected with a low drop-out rate and using well-established and standardized methods. The null findings were consistent in the group of participants with relevant symptoms at the beginning and the full group of all participants, suggesting that the absence of symptom change did not depend on varying levels of baseline symptoms. At the same time, participation was perceived as helpful in overcoming emotional distress, as it allowed the expression of emotions, facilitated social interactions and helped people learn new skills.

There is limited documented evidence about the potential of music education interventions for the management of symptoms of anxiety and depression in adolescents. There is, however, an increasing body of literature focusing on the potential of music-based interventions and active music therapy, some of which include elements of the music workshops studied here, in the management or improvement of such symptoms [19, 28–36].

In this study, we did not find any clinically meaningful or statistically significant symptom changes, which might be due to the short duration of the workshops. The short duration was indeed criticized by several participants, most of whom reported positive experiences and perceived benefits of participating.

The participants’ positive experiences are in line with reports in other studies, such as relationship building, and as a means of self-expression, self-regulation, and self-transformation, the learning of new musical skills such as playing instruments or composing songs allowed participants to see themselves as more capable individuals [35]. In addition, they can improve their ability to cope with negative feelings, improve their self-confidence, reduce anxiety, step outside their comfort zone, rupture their routine, and strengthen their creativity process [28–42].

Although the musical education and practice used in this study did not have a significant effect on symptoms, future research may evaluate other forms of music-based interventions. Despite a wide range of existing psychological and pharmacological treatments, symptom recurrence remains a significant issue in the adolescent population [38, 43], and further interventions are required that help young people engage through their existing interests, provide them with positive ways of developing an understanding of their own mental health [28, 44] and – if possible – equip them with resources to cope with future crises.

### Limitations

The workshops faced logistic difficulties that affected enrolment and attendance. It was difficult to find a venue and a time that would have allowed more participants to attend. Relatedly, the relatively small number of participant and full attendance of all five sessions, which was only 56%, may have limited the potential effects of an intervention that was brief even with full participation.

In this noncontrolled trial, we would not have been able to establish conclusive evidence about its effectiveness. However, the clearly null results suggest that the same approach should not be pursued in future larger and possibly randomized controlled trials.

These workshops were not developed, nor run by, trained music therapists because this intervention hadn´t a therapeutically approach which may have contributed to the null result. However, the interest of our study was to identify the effectiveness of a model of intervention similar to artistic resources available at the community level for young due to the barriers in the access to mental health services in vulnerable settings from low income countries.

Further research might explore interventions using a wider repertoire, including instruments that are more accessible in daily life and based on everyday environments, to facilitate the participation of young people. Additionally, longer and more workshop sessions should be explored, allowing for more learning, which many participants found helpful. Future studies with more participants could also look at the heterogeneity in response to the intervention in addition to using more robust designs.

## Electronic supplementary material

Below is the link to the electronic supplementary material.

Supplementary Material 1

## Data Availability

The consent forms did not specify that the data would be deposited in a public repository, so we cannot deposit the data. The deidentified participant dataset will be made available from the corresponding author () upon reasonable request and subject to a data-sharing agreement.

## Abbreviations

PHQ-8: Patient Health Questionnaire-8
GAD-7: Generalized Anxiety Disorder Assessment Questionnaire-7
INEM: National Institutes of Secondary Education (for its acronym in Spanish).
IED: District Educational Institution (for its acronym in Spanish).
REDCap: Research Electronic Data Capture
