# Supplementary material for: Outcomes and experiences of music workshops for adolescents with depression and anxiety: An exploratory noncontrolled trial in Bogotá
Source: BMC Res Notes. 2024 Dec 2;17:355. doi: 10.1186/s13104-024-07007-z (PMC11613543; doi:10.1186/s13104-024-07007-z)
Supplement: Supplementary file 1 — Supplementary Material 1 [file 13104_2024_7007_MOESM1_ESM.docx]

SUPPLEMENTARY MATERIAL

Table A. Overall attendance

| **Overall attendance per session** | | | | | | |
| --- | --- | --- | --- | --- | --- | --- |
| **Session** | **First** | **Second** | **Third** | **Fourth** | **Fifth** | **Mean** |
| Participants (n=32) | 28 | 27 | 27 | 29 | 27 | 27,6 |
| Percentage | 88% | 84% | 84% | 91% | 84% | 86% |

Table B. Attended sessions

| **Final attended sessions** | | | | | |
| --- | --- | --- | --- | --- | --- |
| **Session** | **One session attended** | **Two sessions attended** | **Three sessions attended** | **Four sessions attended** | **Five sessions attended** |
| Participants (n=32) | 2 (6%) | 0 (0%) | 2 (6%) | 10 (31%) | 18 (56%) |
